# Role of Omega-3 Fatty Acids in Cardiovascular Disease: the Debate Continues

**DOI:** 10.1007/s11883-022-01075-x

**Published:** 2022-12-29

**Authors:** Samuel C. R. Sherratt, Peter Libby, Matthew J. Budoff, Deepak L. Bhatt, R. Preston Mason

**Affiliations:** 1 Department of Molecular, Cellular, and Biomedical Sciences, University of New Hampshire, Durham, NH 03823 USA; 2 Elucida Research LLC, P.O. Box 7100, Beverly, MA 01915-0091 USA; 3 Department of Medicine, Cardiovascular Division, Brigham and Women’s Hospital, Harvard Medical School, Boston, MA 02115-6110 USA; 4 Department of Medicine, Lundquist Institute at Harbor-UCLA Medical Center, Torrance, CA 90502 USA; 5 Mount Sinai Heart, Icahn School of Medicine, Mount Sinai Health System, New York, NY USA

**Keywords:** Atherosclerosis, Triglycerides, Lipoproteins, Omega-3 fatty acids, Cholesterol, Eicosapentaenoic acid

## Abstract

**Purpose of Review:**

The omega-3 fatty acids (n3-FAs), eicosapentaenoic acid (EPA) and docosahexaenoic acid (DHA), have recently undergone testing for their ability to reduce residual cardiovascular (CV) risk among statin-treated subjects. The outcome trials have yielded highly inconsistent results, perhaps attributable to variations in dosage, formulation, and composition. In particular, CV trials using icosapent ethyl (IPE), a highly purified ethyl ester of EPA, reproducibly reduced CV events and progression of atherosclerosis compared with mixed EPA/DHA treatments. This review summarizes the mechanistic evidence for differences among n3-FAs on the development and manifestations of atherothrombotic disease.

**Recent Findings:**

Large randomized clinical trials with n3-FAs have produced discordant outcomes despite similar patient profiles, doses, and triglyceride (TG)-lowering effects. A large, randomized trial with IPE, a prescription EPA only formulation, showed robust reduction in CV events in statin treated patients in a manner proportional to achieved blood EPA concentrations. Multiple trials using mixed EPA/DHA formulations have not shown such benefits, despite similar TG lowering. These inconsistencies have inspired investigations into mechanistic differences among n3-FAs, as EPA and DHA have distinct membrane interactions, metabolic products, effects on cholesterol efflux, antioxidant properties, and tissue distribution. EPA maintains normal membrane cholesterol distribution, enhances endothelial function, and in combination with statins improves features implicated in plaque stability and reduces lipid content of plaques.

**Summary:**

Insights into reductions in residual CV risk have emerged from clinical trials using different formulations of n3-FAs. Among high-risk patients on contemporary care, mixed n3-FA formulations showed no reduction in CV events. The distinct benefits of IPE in multiple trials may arise from pleiotropic actions that correlate with on-treatment EPA levels beyond TG-lowering. These effects include altered platelet function, inflammation, cholesterol distribution, and endothelial dysfunction. Elucidating such mechanisms of vascular protection for EPA may lead to new interventions for atherosclerosis, a disease that continues to expand worldwide.

## Introduction

According to the World Health Organization (WHO), atherothrombotic cardiovascular (CV) disease has become the leading cause of global mortality with a disproportionate impact on low and middle income countries [1]. Despite effective control of low-density lipoproteins (LDL) with oral and non-oral interventions, CV risk persists, likely due in part to elevated triglyceride (TG)-rich lipoproteins (TGRLs), a dyslipidemia particularly prevalent in patients with diabetes and metabolic disease [2, 3, 4•]. Genetic and epidemiologic analyses support an independent role for TGRLs as a risk factor and strong contributor to overall mortality [5, 6, 7••, 8]. TGRL concentrations associate with increased CV risk and their accompanying proteins like apolipoprotein C3 (ApoC3) and angiopoietin-like 3 (ANGPTL3) which limit lipoprotein lipase (LPL) activity, leading to a rise in TGRL levels. Human mendelian randomization studies strongly support the causality of TGRL in atherosclerotic events, but such studies cannot on their own determine the independence of TGs as a risk factor as variants often have pleiotropic effects that have may influence disease etiology [9, 10]. Indeed, TGRL incite inflammation to much greater degree than LDL-C particles, and thus, elevated TGRL may contribute to the inflammatory component of residual risk [11]. Furthermore, agents that effectively lower TGs, including potent fibrate derivatives, have failed to reduce CV events risk in high-risk patients when receiving contemporary care. These findings have engendered debate about the relative atherogenicity of LDL-C cholesterol, measured as apolipoprotein B (ApoB), versus TGs in lipoprotein particles.

Randomized CV trials have tested n3-FAs for residual risk reduction in patients with well-controlled LDL-C levels and elevated TGs. Despite similar and effective TG reductions, the results of these trials have been highly inconsistent. Sufficiently powered outcome trials have demonstrated benefits of an ethyl ester formulation of eicosapentaenoic acid (EPA) known as icosapent ethyl (IPE), but not of more traditional, mixed n3-FA preparations or other TG-lowering agents [12, 13]. These findings have piqued interest in the mechanistic effects of different n3-FA formulations, and especially IPE, in relation to reductions in atherothrombotic risk. In vivo and in vitro studies indicate that EPA has distinct metabolic products, plaque incorporation, membrane interactions, lipid antioxidant activity, and tissue distribution (i.e., arterial) compared to other n3-FAs, especially DHA that concentrates in neuronal membranes [7••, 14]. EPA maintains cholesterol distribution in membranes and preserves normal phospholipid packing constraints, competes with arachidonic acid for cyclooxygenase (COX), and enhances endothelial function in combination with a high intensity statin as compared to DHA [15]. These distinct biological actions for EPA, along with its complex bioactive metabolites, may lead to new insights into mechanisms of atherothrombotic disease and therapeutic interventions.

## Rationale for TG-Lowering and Reduced Residual Risk

Plasma TGs are carried in chylomicrons, VLDLs, and remnant particles, collectively termed TGRLs, and independently predict ischemic events and all-cause mortality [8, 16, 17]. The TGRLs essentially carry all the plasma cholesterol not associated with either LDL-C or HDL in circulation. Data from the Copenhagen General Population Study indicate that elevations in TGs are common in developed countries as over 25% of individuals had TG levels > 176 mg/dL while 21% had remnant cholesterol > 39 mg/dL [8]. Observational studies indicate that individuals with non-fasting TGs of 580 mg/dL have > threefold higher risk for ischemic stroke compared with individuals with levels of 70 mg/dL. Such TG elevations confer a greater than fivefold increased risk for myocardial infarction (MI) and twofold increase in the risk for all-cause mortality that correlated with higher TGRLs.

What mechanisms account for the atherogenicity of TGRLs? Due to their large size, TGRLs do not efficiently penetrate the endothelial layer compared to other lipoprotein particles like LDL. However, smaller “remnant” particles formed from TGRLs during enzymatic catabolism are rich in cholesterol and highly atherogenic. Reduced activity of various lipases that facilitate TGRL catabolism results in excessive remnant formation and increased disease progression. A protein associated with TGRLs, ApoC3, inhibits lipase activity and also associates with increased CV risk [18]. Lipase activity is also inhibited by ANGPTL3 and ANGPTL4 associated with the surface of endothelial cells. These observations have led to new TG-lowering therapies strategies that affect the expression and/or activity of these proteins.

In the recent TRANSLATE-TIMI 70 trial, the ANGPTL3-targeted antisense oligonucleotide vupanorsen reduced TGs in patients with hypertriglyceridemia by more than half compared with placebo [19]. However, the vupanorsen clinical program halted over concerns about efficacy and certain side effects, including liver steatosis [20]. A monoclonal antibody (evinacumab) approach directed against ANGPTL3 effectively reduced LDL-C in patients with homozygous familial hypercholesterolemia and refractory hypercholesterolemia [21, 22]. The antisense oligonucleotide volanesorsen, which decreases ApoC3 expression, has also shown robust TG lowering (77%) in patients with familial chylomicronemia syndrome compared with placebo, but it remains to be seen whether this effect will reduce events in patients with established CV disease risk. [23]

**Fig. 1:**
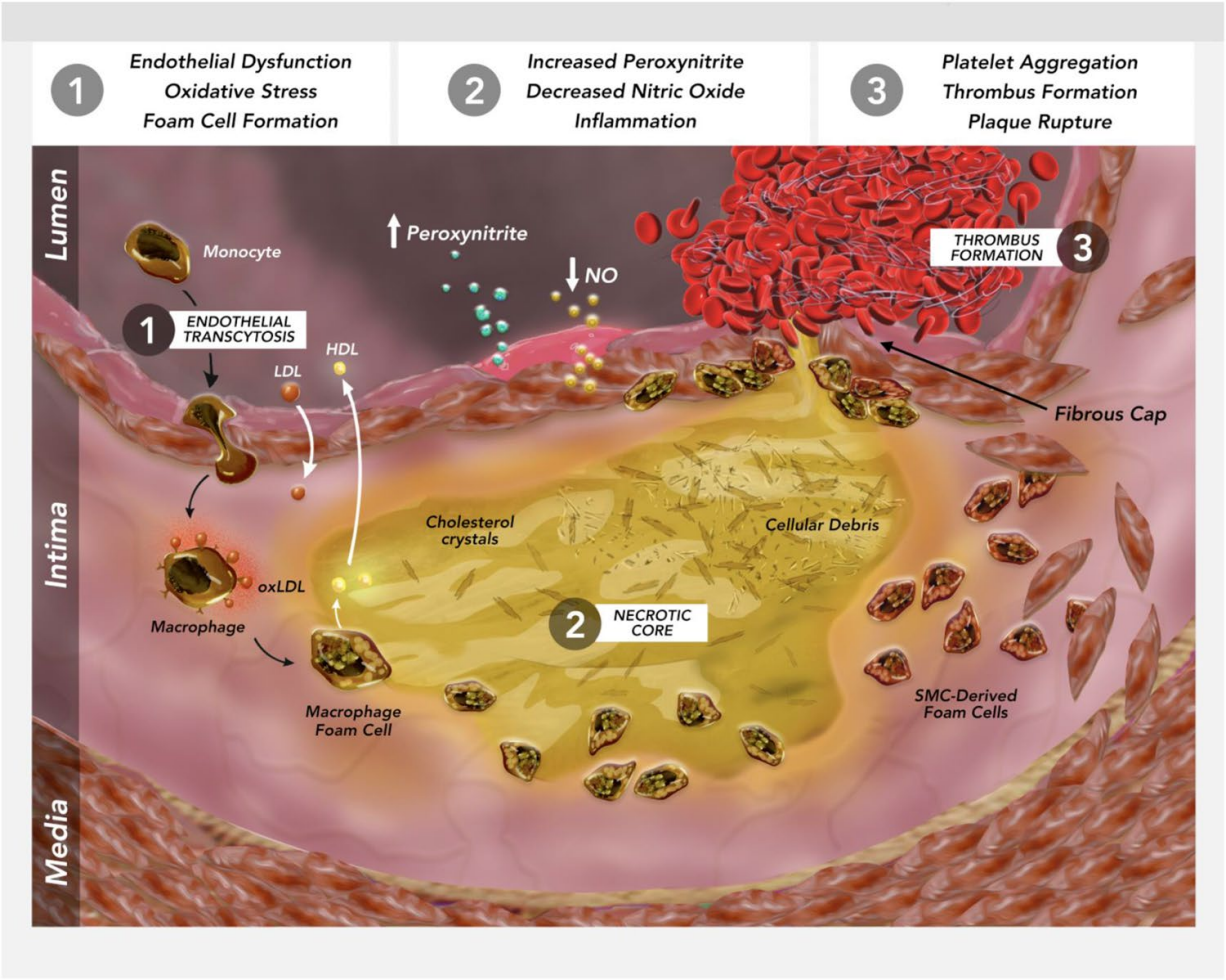
Atherosclerotic plaque initiation and progression—the nexus of lipid accumulation and inflammation. Atherosclerosis is a complex inflammatory response at the nexus of endothelial cell dysfunction, leukocyte activation, lipid accumulation and altered smooth muscle cells functions. If not interrupted, these integrated cellular processes culminate in plaque disruption and formation of an occlusive thrombus

The smaller, atherogenic remnants of TGRLs cross the endothelial layer more efficiently than the larger forms. Within the plaque they promote atherosclerosis and add to the cholesterol content of the lesion. Macrophages efficiently engulf the internalized TGRL remnants to promote foam cell formation in the arterial intima due to their cholesterol content and dependence on internalization by the classical LDL-C receptor. This receptor’s expression falls as intracellular cholesterol rises, rendering foam cell formation difficult by LDL-C loading alone (Fig. 1). As a result, the cholesterol in lesions may depend more on remnants than on other ApoB containing lipoproteins like LDL. The fatty acids associated with TGRLs, particularly saturated fats like palmitate, can activate the NLRP3 (NLR family pyrin domain-containing) inflammasome which, in turn, produces activated forms of the proinflammatory cytokines IL (interleukin)-1β and IL-18 [24]. NLRP3, once activated by various danger signals, recruits the adapted apoptosis-associated speck-like protein containing a caspase recruitment domain (CARD) (ASC). This converts procaspase-1 into caspase-1. The caspase-1 then converts the inactive pro- forms of interleukins (IL)-1β and IL-18 to their mature functional forms for subsequent release from cells [25, 26]. There appear to be several mechanisms of activation for the NLRP3 inflammasome depending on the inflammatory stimulus. Animals deficient in the expression of the NLRP3 inflammasome or cathepsin molecules are unable to mount an inflammatory response to a number of atherogenic stimuli, including cholesterol crystals. [27•]

TGRLs exert direct proinflammatory effects in concert with ApoC3 on macrophages and endothelial cells. The causality of ApoC3 in atherothrombosis is supported by a number of human genetic studies [18]. Rare loss of function mutations that reduce ApoC3 activity were associated with lower risk of CV disease and circulating TGs. Thus, certain protective effects of n3-FAs on atherothrombosis may be due to inhibiting the proinflammatory effects of accumulated TGRLs in concert with reduced ApoC3 levels. [28]

Despite broad epidemiological and genetic evidence associated with elevated TGs and CV risk, clinical trials using TG-lowering agents (e.g., fibrate derivatives, niacin) have failed to reduce such risk [29–31]. Indeed, the extent of TG lowering did not predict changes in CV events in these or other trials using n3-FAs in patients on contemporary care that include statins [13]. These results suggest that the TG content in TGRLs does not causally add to CV risk, rather the cholesterol in these particles may drive their atherogenicity. Indeed, variants associated with these particles suggest that changes in ApoB absolute levels were the best predictor of CV event reduction after controlling for other variables using mendelian randomization analyses [10]. Thus, TG-lowering itself may not provide incremental protection for high risk patients who receive sufficient LDL-C lowering therapy. [12, 13]

A trial was conducted using a fenofibrate derivative known as a selective peroxisome proliferator-activated receptor alpha modulator (SPPARM-α). This compound, pemafibrate, was selected as it has greater affinity and specificity for PPAR-α by more than 2000-fold compared to either PPAR-γ or -δ (delta). After promising phase 2 trials, The Pemafibrate to Reduce Cardiovascular Outcomes by Reducing Triglycerides in Patients with Diabetes (PROMINENT) trial prospectively enrolled patients with elevated TGs as subgroups of such patients had previously showed the greatest potential for benefit with effective TG-lowering agents [32]. The primary objective of this phase 3 study was to test the ability of pemafibrate (0.2 mg bid) to delay the time of the first occurrence of nonfatal myocardial infarction (MI), nonfatal ischemic stroke, hospitalization for unstable angina requiring unplanned coronary revascularization, and CV death in statin treated patients with diabetes and moderate hypertriglyceridemia (fasting TGs: ≥ 200 to < 500 mg/dL; HDL-C ≤ 40 mg/dL). PROMINENT was terminated early in these high risk patients due to futility following the recommendation of the independent data-monitoring committee [33]. Thus, TG-lowering agents from different classes and potency have consistently failed to reduce CV risk on top of high intensity statins despite effective TG lowering. [29–31]

## The n3-FAs Reduce TGs Through Various Mechanisms

The n3-FAs effectively lower TGs in a dose-dependent manner by enhanced fatty acid oxidation as well as blockade of acyl-CoA:1,2-diacylglycerol acyltransferase (DGAT), resulting in attenuated hepatic VLDL production and lipogenesis [34, 35]. The n3-FAs specifically stimulate the G-protein coupled receptor GPR120, promoting brown and beige adipocyte differentiation, thus producing thermogenic activation [36–38]. GPR120 binding also causes the release of fibroblast growth factor-21 (FGF21) by adipocytes. FGF21 knock out in mice impairs GPR120-mediated adipocyte activation and browning. Thus, GPR120 activates adipocytes by a mechanism that involves induction of FGF21 following stimulation with n3-FAs. [37]

EPA and other n3-FAs like DHA are also especially effective fatty acid agonists of PPARs [39]. PPARs are part of the nuclear hormone receptor family and represent a subgroup of three ligand-inducible transcription factors. Three different isoforms of PPARs have been described so far in mammals: PPAR-α, PPAR-β/δ and PPAR-γ. These are part of a nuclear hormone receptor superfamily and, by binding to PPAR-responsive regulatory elements (PPRE), heterodimerize with the retinoid X receptor (RXR). This binding leads to formation of an active transcriptional complex that regulates various genes involved in lipid metabolism. This activated complex also regulates various aspects of adipogenesis, inflammation and metabolic homeostasis [40]. PPAR-α activation reduces VLDL and TG levels while increasing circulating high density lipoprotein (HDL) levels following induction of hepatic apolipoprotein A-I and apolipoprotein A-II expression. Fibrates (PPARα agonists) lower circulating TG levels while glitazones (PPAR-γ agonists) induce lipoprotein lipase expression in adipose tissue. [41]

Circulating TGs correlate strongly with plasma apolipoprotein levels in patients with hypertriglyceridemia. In the MARINE and ANCHOR trials, IPE treatment decreased levels of ApoC3 levels along with TGs, resulting in improved LPL activity and endocytosis of ApoB particles [28]. Certain n3-FA derived N-acyl taurines (NATs) also inhibit TG hydrolysis and absorption in enterocytes from animals fed a high fat diet [42]. Thus, the multiple mechanism(s) of TG-lowering for IPE include changes in lipogenesis, beta oxidation, lipase activity and gene expression.

## High-Dose EPA Treatment and Residual Cardiovascular Risk

In contrast to previous TG-lowering trials, IPE reduced a composite of CV disease events in such patients with elevated TGs. REDUCE-IT investigated the effects of IPE on residual CV disease risk in statin treated patients [43••]. The trial randomized over 8,000 patients with elevated TGs (≥ 135 and < 499 mg/dL) and established CV disease or diabetes with at least one additional risk factor to 4 g/d IPE or mineral oil placebo. This multicenter, placebo-controlled trial enrolled approximately 71% of the participants based on secondary CV prevention while the remaining subjects (29%) had diabetes and at least one other risk factor. The primary endpoint was a 5-point composite MACE (CV death, non-fatal MI, non-fatal stroke, hospitalization for unstable angina, and coronary revascularization). REDUCE-IT showed a 25% relative risk reduction (HR: 0.75, 95% confidence interval (CI): 0.68–0.83, *P* < 0.001) and 4.8% (95% CI: 3.1–6.5) absolute risk reduction of the primary endpoint with a number needed to treat (NNT) of 21.

At the first prespecified interim analysis, the Data Safety Monitoring Board for REDUCE-IT detected a risk reduction of the primary endpoint with IPE (HR: 0.77, 95% CI: 0.68–0.87, *P* < 0.001) after approximated 60% expected events had occurred. This reduction in events achieved significance after approximately 21 months following randomization, indicating an early benefit with IPE treatment [44]. Prespecified hierarchical analysis of endpoints showed that IPE treatment lowered risk of fatal or non-fatal MI by 31% (*P* < 0.001), fatal or non-fatal stroke by 28% (*P* = 0.01), and CV death by 20% (*P* = 0.03). Total ischemic events (first and subsequent) fell by 30% (*P* < 0.001) in the IPE treatment arm, and first coronary revascularizations declined by 34% (*P* < 0.001) [45, 46]. Both post hoc and prespecified subgroup analyses revealed consistent event risk reduction with IPE regardless of CV disease history, including patients with prior MI (26%, *P* < 0.001), prior percutaneous coronary intervention (PCI, 34%, *P* < 0.001), prior coronary artery bypass graft (CABG, 24%, *P* = 0.004), or with varying kidney function based on estimated glomerular filtration rate (eGFR, < 60 mL/min (29%, *P* < 0.001), ≥ 60 and < 90 mL/min (20%, *P* = 0.001), and ≥ 90 mL/min (30%, *P* = 0.003). [47–50]

Additionally, although all patients were enrolled based on adherence to statin therapy, the risk reduction with IPE did not differ based on the type of statin (lipophilic or lipophobic) the patients received [51]. Finally, there was a 31% relative risk reduction in the primary composite endpoint in the 3,146 patients enrolled in the USA (*P* < 0.001) [52]. Importantly, despite the baseline TG threshold required for enrollment in REDUCE-IT, IPE conferred a consistent risk reduction regardless of baseline or achieved TG levels, indicating that TG lowering itself does not explain the mechanism of event reduction in IPE-treated patients. [43••]

IPE treatment was associated with more serious adverse events in REDUCE-IT but similar to placebo. In particular, there were increased events related to hospitalization for atrial fibrillation (3.1% for EPA vs 2.1% for placebo, *P* = 0.004) [43••]. However, this was not clinically significant as there were substantially lower rates of stroke with IPE (28% reduction in fatal or non-fatal stroke, *P* = 0.01). Bleeding rates overall were also low but slightly higher in subjects treated with IPE (2.7% vs 2.1%, *p* = 0.06), with no fatal bleeding events related to study drug. There was also no significant increase in gastrointestinal bleeding or hemorrhagic stroke events in adjudicated cases.

Favorable outcomes with IPE were also reported in the Japan EPA Lipid Intervention Study (JELIS) trial. JELIS enrolled subjects with hypercholesterolemia but not a pre-specified minimum TG level [53•]. This was an open label trial where 18,645 patients were randomized to IPE (1.8 g/d) on top of a statin. IPE treatment was associated with a 19% (*p* = 0.011) reduction in CV events compared to subjects administered statin alone. The population in the JELIS study included both primary and secondary prevention participants. The median plasma TG levels of the participants at baseline (153 mg/dL) were close to normal and TG levels fell only 9% overall, compared to baseline with IPE treatment. Again, TG lowering with IPE treatment did not predict event reduction as observed in REDUCE-IT. A post hoc review in JELIS subjects with elevated TG levels (> 150 mg/dl) at baseline along with low HDL-C levels (< 40 mg/dl) showed a 53% reduction (*p* = 0.043) in events with IPE treatment. [54]

## High-Dose EPA and Progression of Atherosclerosis in Patients with Elevated TGs

The benefits with IPE beyond TG lowering may indicate direct effects of EPA on progression of atherosclerosis not reproduced with low dose or mixed n3-FA preparations that include DHA in the formulation [12, 13]. Indeed, the best predictor for outcomes with IPE in REDUCE-IT was blood EPA concentrations as compared with other biomarkers of CV risk [55]. Imaging studies indicate that EPA has direct effects on plaque progression in patients with coronary artery disease. Integrated backscatter intravascular ultrasound (IB-IVUS) was used to measure changes in coronary thin-cap fibroatheroma in IPE treated patients versus statin alone in the Combination Therapy of Eicosapentaenoic Acid and Pitavastatin for Coronary Plaque Regression Evaluated by Integrated Backscatter Intravascular Ultrasonography (CHERRY) trial [56]. The IPE plus statin intervention was associated with a significant reduction in plaque volume concomitant with a decrease in the AA-EPA ratio compared with statin alone. A similar randomized trial of IPE plus statin versus statin alone found a significant increase in fibrous cap thickness with IPE plus statin therapy versus statin therapy alone using optical computed tomography (OCT). [57]

The randomized Effect of Vascepa on Improving Coronary Atherosclerosis in People with High Triglycerides Taking Statin Therapy (EVAPORATE) trial used multidetector computed tomography to measure the effects of IPE in statin-treated patients having coronary disease and dyslipidemia compared to statin alone [58•]. IPE (4 g/day) treatment for 18 months correlated with reduced low attenuation plaque (LAP) volume by 17% compared with baseline. This may be particularly important in explaining the reductions of MI with IPE, as patients with a LAP burden of more than 4% are more likely to experience an MI [59]. Plaque regression with intervention in EVAPORATE was consistent in several measurements of plaque volume including total plaque. Changes in vulnerable plaque characteristics with IPE treatment was not associated with TG or other lipid changes. The benefits of IPE were observed even after multivariable adjustment for risk factors of CV. [58•]

These changes in plaque volume and fibrous cap thickness with IPE treatment correlate with changes in the cellular content of plaque. The n3-FAs, along with their various metabolites, differentially associated with lipid-rich plaques in atherosclerosis prone animals. In ApoE-deficient mice fed a Western diet, mass spectrometry measured the plaque distribution of n3-FAs and their metabolites, in conjunction with histological analysis [60•]. Along with a common metabolite, 12-hydroxy-EPA, EPA was associated with thin-cap plaques and an increase in M2 macrophages. A concentration gradient for EPA that extended from the endothelium to the media was also observed in the aortic arch. Remarkably, DHA associated randomly with both thin- and thick-cap plaques while not affecting intima-media thickness; EPA, by contrast, significantly reduced plaque thickness.

Additional insights into the arterial distribution of EPA versus DHA came from carotid endarterectomy studies in patients administered a short-term intervention of mixed formulation n3-FAs [61]. Once again, a preferential association of EPA but not DHA with lipid rich lesions was observed in the endarterectomy. There was also an inverse correlation between indicators of plaque instability, T cell number and overall inflammation with the amount of phospholipid-containing EPA. EPA incorporated into atherosclerotic lesions where it modified indices of tissue inflammation, including T cell content as well as foam cells. Collectively, these results show consistent effects on plaque stability with EPA to a greater extent than other n3-FAs like DHA. These findings may help explain the broad effects of IPE treatment on atherothrombotic-related event risk reduction in large clinical trials.

## Mixed Omega-3 Fatty Acid Treatments and Residual Cardiovascular Risk

At the same or lower doses, mixed n3-FA formulations containing EPA and DHA have not reproduced the clinical benefits of IPE reported in large outcome trials. The Long-Term Outcomes Study to Assess Statin Residual Risk with Epanova in High Cardiovascular Risk Patients with Hypertriglyceridemia (STRENGTH) trial evaluated the effects of a carboxylic acid formulation of EPA and DHA (4 g/d) in 13,078 patients with elevated TGs and CV disease risk including diabetes, quite similar to the REDUCE-IT population [62•]. Similar to previous mixed n3-FA formulations, this well-conducted trial did not show any reduction in risk of CV events. The STRENGTH trial was thus halted prematurely despite a 19% reduction in TG levels. This result, along with other trials discussed below, raises questions about possible distinct biological effects of DHA that counter the beneficial effects of EPA.

The null findings from STRENGTH were consistent with the Study of Cardiovascular Events in Diabetes (ASCEND) trial investigating the effects of n3-FA supplementation for primary prevention of CV events in patients with diabetes [63]. Specifically, this trial tested mixed EPA/DHA at the lower dose of 1 g/d on the incidence of serious vascular events in over 15,000 patients with diabetes but not atherosclerotic disease and, importantly, without a statin adherence requirement. There was no reduction in first serious vascular events among subjects randomized to treatment. Similarly, The Vitamin D and Omega-3 Trial (VITAL) tested a mixed n3-FA formulation at 1 g/d (and vitamin D_3_ at 2,000 IU/d) in close to 26,000 patients, including more than 5,000 black patients, for primary prevention of CV events and invasive cancer [64]. Again, neither the composite CV endpoint nor cancer-associated endpoint fell significantly. Thus, STRENGTH, ASCEND and VITAL showed no benefit in either primary and secondary CV populations using various mixed EPA/DHA formulations and doses. Several meta-analyses cast further doubt on the benefits of mixed n3-FA formulations for CV risk reduction, routinely showing no significant reduction of major CV events. [65•, 66–68]

Blood levels of EPA emerged as the best predictor of clinical benefit in trials using IPE such as JELIS and REDUCE-IT [55]. Remarkably, a similar relationship was not reported in trials that used mixed n3-FA formulations, including STRENGTH, despite the highest tertile of achieved plasma EPA levels (151 µg/mL) reaching similar levels to the median serum EPA levels in REDUCE-IT (144 µg/mL) [69]. Part of the explanation may be that changes in EPA always associated with concomitant increases in DHA (median for highest tertile of achieved plasma DHA levels in STRENGTH was 118 µg/mL), a fatty acid that competes with EPA for similar enzymatic pathways and has nearly opposite effects on membrane interactions and cholesterol distribution [70]. In the OMega-3 fatty acids in Elderly with Myocardial Infarction (OMEMI) trial, an EPA/DHA prescription formulation (1.8 g/d) was tested in patients with a recent acute MI [71]. The EPA/DHA treatment did not reduce the primary endpoint of CV events even as median EPA (87%) levels increased. In this trial, the increase in EPA was again accompanied by a significant increase in serum levels of DHA (16%) [71, 72]. A minimum or threshold level of EPA may be required for meaningful CV risk reduction in outcome trials using n3-FAs. In STRENGTH, the overall median on-treatment EPA levels were low, even as compared with the baseline levels of measured in the JELIS trial (89.6 vs. 97 µg/mL, respectively), and were 40% less than in subjects enrolled in REDUCE-IT [7••, 70]. Additionally, the chemical form of the fatty acids in each formulation may contribute to differences in absorption, distribution and ultimately clinical outcomes (ethyl ester in REDUCE-IT, carboxylic acid in STRENGTH), though this requires further investigation.

It has been argued that the CV benefit with IPE in certain trials (most notably REDUCE-IT) related to the use of pharmaceutical grade mineral oil as comparator. Some posit that mineral oil may increase CV risk directly or indirectly by altering metabolism of other drugs even at the dose used in REDUCE-IT (2 g bid), which was almost tenfold less than the amount used in treatment of constipation (30–90 g). A post hoc analysis of REDUCE-IT showed that LDL-C, IL-1β, and hsCRP levels increased significantly in the placebo arm at 12 months compared with baseline (hsCRP 2.8 mg/L vs. 1.8 mg/L; LDL-C 96 mg/dL vs 87 mg/dL; IL-1β 0.06 pg/mL vs 0.08 pg/mL) [43••, 73]. While these changes were statistically significant, it is unclear if they convey clinical significance to such a degree as to negate the benefits of IPE in clinical trials. When placed in context with other CVOTs, the changes in inflammatory biomarkers appear negligible. For example, the baseline hsCRP levels in the JUPITER trial were almost double the baseline levels in REDUCE-IT (4.3 vs 2.2 mg/dL), and baseline IL-1β levels in the CIRT trial were more than 24-times greater than baseline levels in REDUCE-IT (1.46 vs 0.06 pg/mL) [74, 75]. Based on these values, it is clear that the patient population in REDUCE-IT was distinct from those in other CVOTs recruiting for anti-inflammatory treatments, and thus, not surprising that event reduction with IPE did not correlate with reductions in these markers. Additionally, if mineral oil were causing pro-inflammatory effects to an extent that would artificially indicate event reduction with IPE, one might expect that the inflammatory biomarkers would continue to increase with continuous administration. However, the hsCRP and IL-1β levels in the placebo arm do not significantly increase after 12 months through the end of the observation period. To adequately determine if the mechanism of action of IPE involves the IL-1-IL-6-hsCRP axis, a prospective, randomized controlled trial that recruits patients based on elevated hsCRP levels would be required. Additionally, a comprehensive review of mineral oil use in CV trials found no reproducible, consistently statistically significant effect of mineral oil on inflammatory markers, including hsCRP, or lipid levels, challenging a mechanistic argument that mineral oil contributed to these biomarker changes. [76]

It has also been proposed that mineral oil, despite very low oral absorption, interferes with the pharmacokinetics of statins, leading to reduced potency and LDL-C reductions. Animal experiments tested this conjecture directly with lipophilic and hydrophilic statins, including atorvastatin and pravastatin, respectively. [77] Administered levels of mineral oil that reproduced REDUCE-IT resulted in no change in the absorption and bioavailability of these statins compared with water placebo. Indeed, there was also no difference in beneficial outcomes among patients in REDUCE-IT being administered either lipophilic or hydrophilic statins [77]. One would expect mineral oil to introduce a bias against certain hydrophobic statins based on their affinity to oil, but this was not observed in either laboratory animals or in REDUCE-IT. They also measured and found that typical inflammatory markers were not affected on days 10 and 22 in repeat daily dosing groups that received atorvastatin in the absence and presence of mineral oil, including plasma levels of IL-5, IL-6, IL-10, and tumor necrosis factor-α (TNF-α). There were also no significant differences in the mean number of T cells, macrophages or B cells in either the small intestine or colon after repeat dosing of atorvastatin with or without mineral oil co-administration.

Post hoc analyses performed independently by the FDA demonstrated that the benefits of IPE were not influenced by changes in LDL-C levels in subjects randomized to mineral oil or with increases in hsCRP [76, 78]. Several other biomarkers that correlate with LDL-C and hsCRP showed similar patterns, though in many cases, the changes were large on a relative scale, but small on an absolute scale and many were below the lower level of quantification of the assays [73]. The FDA thus concluded that the choice of placebo would have minimal impact, if any, on the clinical outcomes in REDUCE-IT [76, 78]. Finally, computed tomography analysis of the effects of mineral oil on total and non-calcified plaque volume showed no differences in progression from baseline as compared to non-mineral oil comparators consisting of cellulose. [79]

## Do Fish Oil Supplements Have a Role in Reducing Cardiovascular Disease?

The n3-FAs EPA and DHA must be obtained through the diet as humans lack the necessary enzyme for adding the ω-3 double bond (Δ15 desaturase) to long chain fatty acids [80]. Dietary sources of n3-FAs include marine oily fish that, in turn, obtain these oils though their consumption of marine algae and plankton. Additionally, there are plant sources for α-linolenic acid (ALA, 18:3 n-3), a precursor to the more unsaturated and longer chained EPA and DHA. ALA can be obtained from the oil of various plant seeds and nuts including flaxseed, chia, walnuts and echium seeds [81]. Only a small percentage of ALA is converted by humans to EPA or DHA, except among pre-menopausal women where elevated estrogen levels promote the conversion of ALA to DHA, a fatty acid necessary for fetal development. [82–84]

Levels of n3-FAs vary widely among fish species depending on their diets and metabolism. Marine species like mackerel or salmon have up to 4 g per serving as compared with less oily white fish. For instance, tilapia has tenfold less tissue levels of n3-FAs while instead enriched with n6-FAs [85]. Even in fish typically rich in n3-FAs, these levels will decrease if natural sources of algae are replaced by commercial food sources including vegetable-based grains that contain n6-FAs (e.g., linoleic acid) [86, 87]. Thus, fish consumption alone may not provide adequate n3-FA content to elicit the benefits for CV health. Another popular option for patients to increase n3-FA intake is fish oil dietary supplements (FODS) and krill oil supplements [88]. These contain sources of n3-FAs primarily in the form of TGs as found in the tissue of most marine species, while in krill a disproportionate amount of EPA and DHA is associated with phospholipids (30–65%) [89]. The interest in FODS recommendations by the American Heart Association (AHA) that promote regular consumption of fish as part of a healthy diet. [90]

The FODS products do not require clinical testing or rigorous manufacturing like prescription and over the counter preparations. This has led to concerns about the advertised content of FODS and chemical integrity of the oils found in these manufactured products [91]. Due to FDA oversight, pharmaceutical offerings have highly purified formulations of n3-FAs with highly consistent amounts of EPA and/or DHA. But in FODS, there are a plethora of oils beyond n3-FAs that may not be healthy [92]. In a typical FODS product, only one-third of the total oil content is actually n3-FAs. Patients would need to take > 10 capsules to achieve a therapeutic dose (up to 4 g/day) of mixed n3-FAs. Even more capsules would be needed to achieve adequate levels of EPA alone. At least thirty different FAs in leading FODS have been identified and this includes many different medium and long chain saturated fatty acids, which are high in caloric content and not recommended for patients with high CV risk [92]. Primary and secondary products of oxidation were also measured in leading FODS (by sales). All of the FODS had levels of oxidized constituent products that exceeded those considered acceptable for human consumption by the US Council for Responsible Nutrition. By contrast, an n3-FA prescription product had no significant levels of oxidation products or other unhealthy oils (e.g., saturated fats).

The majority of the n3-FAs and other fatty acids used for common FODS is obtained during protein isolation from oily marine fish during industrial manufacturing of agricultural feed products [93, 94]. The n3-FAs, in particular, that are isolated during this extraction procedure are highly susceptible to oxidative damage due to multiple and conjugated unsaturated double bonds. Such fish tissue separation and oil extraction also involves exposure to very high temperatures [94]. There is also exposure to light and contaminants, leading to substantial oxidative damage to the n3-FAs and other unsaturated long chain FAs that are used in these products. In controlled experiments, such oxidative modification of these fatty acids resulted in nearly total loss of antioxidant function and other biological properties associated with its safety and mechanism of action. [95, 96]

Widely published studies from independent laboratories have confirmed these reported discrepancies in the content, purity and rancidity of FODS [92, 97•, 98, 99]. Investigations funded by the US Department of Agriculture showed that only ten out of a total 47 FODS products had accurate EPA levels based on advertised amounts [99]. Additionally, over 70% of these supplements contained less levels of total n3-FAs than promoted. A group from the University of Auckland reported that only 9% of FODS tested had n3-FA levels consistent with their labeled amounts and that the majority (80%) had elevated levels of lipid peroxides, a primary chemical product of oxidation [98]. Similar findings were reported for FODS obtained in North America with respect to lipid oxidation content and loss in biological activity [92, 100]. Together, these data indicate the unsuitability of FODS as alternatives for patients compared with direct fish intake or prescription formulations. FODS are especially inappropriate for patients at elevated CV disease risk in whom high doses of purified EPA are required for risk reduction.

## Lipid Metabolites from Omega-3 Fatty Acids Reduce Inflammation and Promote Homeostasis

Cells incorporate n3-FAs into phospholipids in the endoplasmic reticulum before they are incorporated into membranes during their synthesis. The ester linked n3-FAs are especially abundant in the plasma membrane of different tissues, including retina and neuronal membranes. Phospholipase A_2_ (PLA_2_) can then release the n3-FAs from these phospholipids to be used as substrate for the generation of specialized pro-resolving mediators (SPMs) by cytoplasmic COXs, lipoxygenases (LOXs), and cytochrome P450 (CYP) [101, 102•]. Additionally, COXs, and LOXs can also produce pro-inflammatory (e.g. leukotriene B_4_) and anti-inflammatory (e.g. lipoxin A_4_) eicosanoids from n6-FAs like AA [103]. Compared with EPA, AA is more selective for COX enzymes, especially following acetylation by aspirin which yields production of specific aspirin-triggered anti-inflammatory lipoxins (ATLs) [104]. Platelet activators and other contributors to atherothrombosis like thromboxane A2 also derive from n6-FAs [105]. In competing for the same COX enzymes, n3-FAs reduce pro-aggregatory thromboxanes and vasoconstrictors by producing thromboxane A3 and prostaglandin I3 (PGI3) [106, 107]. Prostacyclin derived from EPA, DPA and DHA specifically inhibit platelets while promoting vasodilation through endothelial-dependent NO release.

To restore homeostasis during acute and chronic disease, including atherosclerosis, the n3-FAs generate the pro-resolving mediators maresins, protectins, and resolvins, which collectively comprise the class of SPMs in macrophages and neutrophils from LOXs (we refer readers to the review by Serhan and Levy from 2018 for a comprehensive summary figure of SPMs and their synthetic pathways) [102•, 108]. Imbalances in the ratio of SPMs and pro-inflammatory lipid metabolites have been associated with unstable plaque features in human carotid endarterectomy specimens and in experimental atherosclerosis [109, 110]. SPMs resolve inflammation through a myriad of mechanisms including reducing granulocyte trafficking and cytokine generation, along with removal of cellular damage by macrophages [111, 112]. There are also changes in the balance of T helper type 1 (Th1) cell to T helper type 2 (Th2) along with polarization of CD4 + T-cells toward a Th2 identity [113–115]. The SPM family derived from EPA includes resolvin E1 (RvE1) strongly implicated in restoring tissue homeostasis. RvE1 can block T-cell activation, Th17 cell stimulation and chemoattraction, further promoting the resolution of inflammation. [116]

## Comparative Biophysical and Antioxidant Properties of EPA and DHA

The n3-FAs concentrate in cell membranes where they modulate structure and membrane lipid rafts while also being metabolized into various bioactive lipids within the cytoplasm. In particular, DHA has additional carbon atoms and one double bond compared with EPA and thus generates different SPMs. The use of independent biophysical approaches recently demonstrated that EPA and DHA have opposing effects on phospholipid interactions and cholesterol distribution in isolated model membranes in vitro. [117••, 118••] EPA maintained intermolecular phospholipid packing constraints and preserved the even distribution of cholesterol while DHA led to disordering of membrane phospholipids, thus causing cholesterol to self-aggregate [118••, 119•]. An equimolar combination of EPA and DHA essentially eliminated their distinct effects, such that there was only a small change in membrane structure when combined [117••]. This direct biophysical evidence supports counter-regulatory actions of EPA and DHA in cell function and membrane biology.

The disparate effects of EPA and DHA on cholesterol-dependent, membrane physical properties were recently confirmed using micropipette aspiration techniques [118••]. This approach allows for the controlled application of force to the membrane surface to facilitate measurements of the apparent expansion modulus (K_app_), a surrogate for membrane lipid elasticity. In isolated membranes containing normal levels of cholesterol, DHA reduced the K_app_ and promoted cholesterol domain formation in membranes. By sequestering cholesterol from bulk lipid, DHA reduces van der Wall interactions between lipid constituents. As a result, DHA reduces the energy required to stretch between membrane cholesterol and phospholipid acyl chain segments. In contrast, EPA promoted an even, homogenous distribution of membrane cholesterol as characterized by a higher K_app_ value.

Fluorescence polarization techniques have substantiated the counter regulatory effects of EPA and DHA on membrane dynamics [119•]. Changes in membrane fluidity were measured in the absence or presence of EPA and DHA by monitoring the apparent rotational correlation time (ARCT) of the molecule, 1,6-diphenyl-1,3,5-hexatriene (DPH) which is a lipophilic florescent probe. DHA increased the ARCT in a significant and dose-dependent manner, while EPA had no effect over a broad range (1–10 mol%) of concentrations. X-ray diffraction analysis of these same membrane preparations confirmed the presence of distinct domains enriched in cholesterol with DHA only. These data indicate that DHA, unlike EPA, promotes membrane cholesterol aggregation due to its inherent fluidizing effects. 2H NMR spectroscopy studies have shown that the same cholesterol-repelling effects with DHA due to rapid isomerizations on a nanoscale time frame [120, 121]. There are multiple examples in biology of closely related molecules having distinct physiologic actions, metabolism and tissue distributions. The sex hormones, in particular, have actions that differ dramatically despite sharing many similar chemical features. Estradiol differs from testosterone only slightly, with one less methyl (CH_3_) group at the 19-carbon position and the carbonyl (C = O) group at the C-3 position being reduced to an alcohol (OH).

EPA has distinct effects on membrane proteins involved in sterol transport compared to DHA, an important mechanism of atheroprotection. The contrasting effects of these n3-FAs were measured in human THP-1 macrophages. Specifically, Dakroub and colleagues measured ABCA1 transporter activity and cholesterol efflux to extracellular acceptors. This is the primary mechanism for cholesterol transfer from peripheral tissue to the liver [122]. Unlike DHA, EPA increased reverse cholesterol transport and ABCA1 activity in these cells. The actions of EPA were independent of changes in ABCA1 membrane density or cell phenotype. EPA treatment also increased the EPA/AA ratio and docosapentaenoic acid (DPA, 22:5, n3) levels unlike DHA. Similar changes in fatty acid composition, including increased EPA/AA ratio and DPA levels, were also reported with EPA treatment in human ECs. [123]

EPA and DHA have distinct tissue distributions that provide insights into difference in metabolism and function. DHA is abundant in neuronal and retinal membranes where it represents the most common polyunsaturated fatty acid and making up to half of total such lipids. In the brain, the conformational changes of DHA promote and stabilize lipid rafts enriched with cholesterol and sphingomyelin [124, 125]. The rapid motion of DHA also provides the necessary fluid environment for proteins such as rhodopsin involved in signal transduction which undergoes changes conformation during photon excitation [126]. In contrast to DHA, EPA concentrates in atherosclerotic plaque when administered at pharmacologic doses where it competes with AA for COX, thereby favoring release of certain anti-inflammatory mediators and increasing the EPA/AA ratio. [7••, 60•]

## Effects of Omega-3 Fatty Acids on Membrane Lipid Oxidation and Cholesterol Crystal Formation During Hyperglycemia

Membrane lipid peroxidation promotes cholesterol domains and reductions in its molecular width as a consequence of lipid peroxyl radicals that cause acyl chain damage and eventual cleavage [127–130]. Due to their multiple double bonds, n3-FAs can stabilize or trap free radicals in resonance structures in the hydrocarbon core where they are incorporated into membrane phospholipids. These molecules are themselves vulnerable to oxidation unless the free radicals are donated to water soluble electron acceptors like ascorbate. At elevated levels, EPA exhibited sustained antioxidant properties as evidenced by reduced lipid oxidation in various ApoB-containing lipoproteins under disease-like conditions [119•, 131, 132•, 133]. By contrast, TG-lowering agents like fibrates, niacin and other n3-FAs had little if any activity in both lipoproteins and membranes in vitro. The radical neutralizing property of EPA depends on its five double bonds: removal of two double bonds (eicosatrienoic acid, 20:3) shows a nearly 50% reduction in antioxidant activity [132•]. Long chain fatty acids with two or fewer double bonds exhibit no antioxidant protection due to the absence of resonance stabilization properties.

EPA significantly reduced circulating levels of oxidized LDL-C as compared with placebo alone in patients with moderate or very high TGs [134]. DHA can exert some antioxidant effects, but these actions diminish with time due to the inherent rapid *trans-gauche* isomerization that leads to disruptions in adjacent acyl chains at the *sn-*1 position and inefficient inhibition of lipid peroxyl radical propagation [117••, 120, 132•, 135–137]. The antioxidant effects of EPA increase when combined with the atorvastatin active metabolite, which occupies a common membrane location to facilitate further resonance stabilization of reactive oxygen species (ROS) through its aromatic ring structures embedded in the membrane lipid bilayer. [127, 133, 138, 139]

**Fig. 2:**
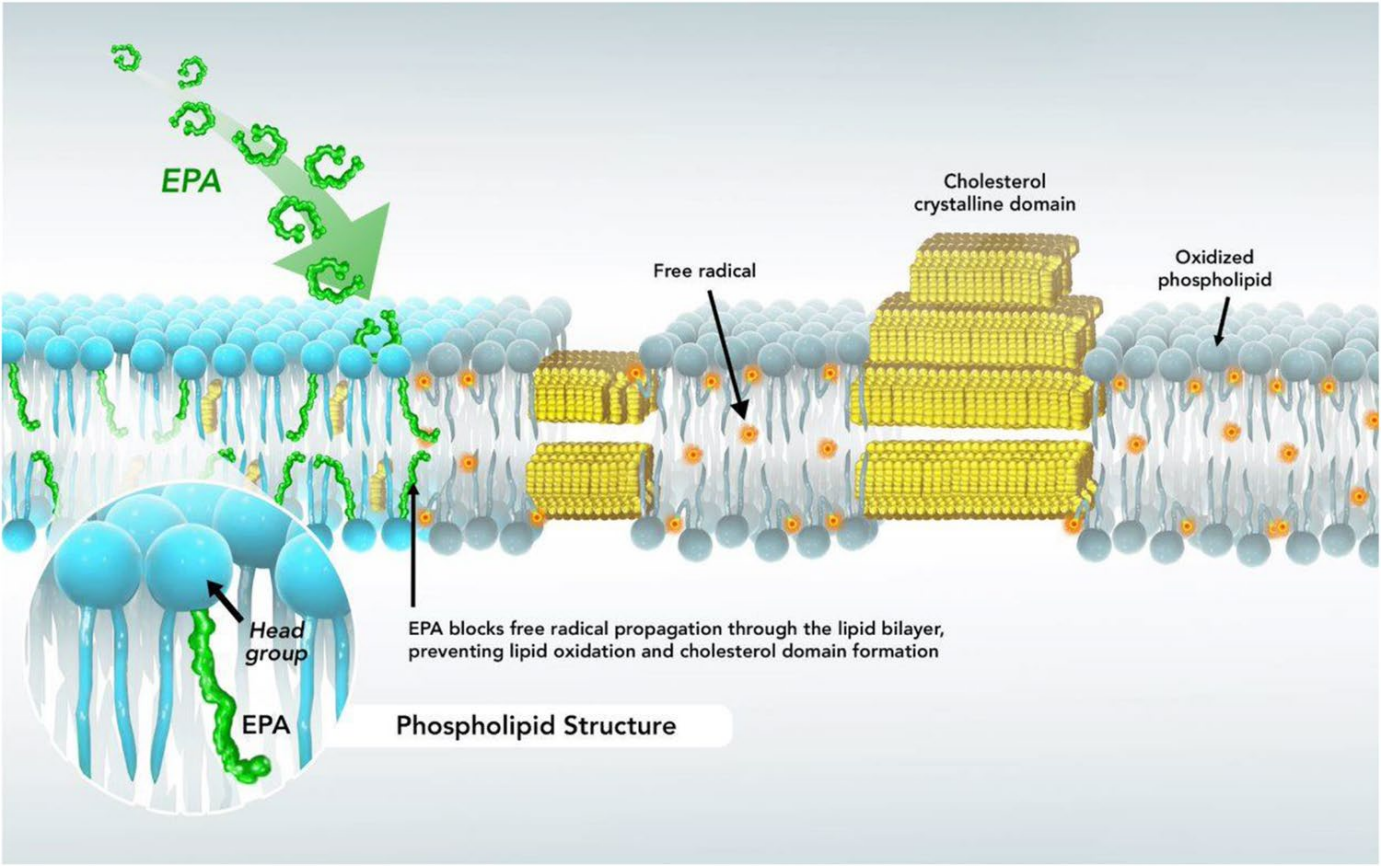
EPA incorporates into phospholipids of membrane bilayers and interrupts the propagation of oxygen free radicals and prevents cholesterol domain formation. Circulating EPA enters cells and is esterified during the biosynthesis of membrane phospholipids. Following incorporation into plasma membrane, EPA has a stable, extended conformation within the membrane that inhibits the propagation of lipid peroxyl radicals and consequent cholesterol crystal formation

The lipid antioxidant effects of EPA were also observed in membranes under disease-like conditions of hyperglycemia. High glucose levels independently increase ROS production and the formation of carbonyl species and other oxidation products associated with lipid degradation [129, 131]. Such effects promote discrete changes in lipid order and structural properties that are manifested by increased cell permeability and loss of barrier integrity [14, 140]. Prolonged exposure to elevated glucose also promotes membrane cholesterol domain formation that precipitate extracellular crystals and their eventual deposition in the necrotic core of the atheroma [129, 131]. The ability of glucose to promote lipid oxidation and cholesterol crystals appear selective, as other monosaccharides (e.g., mannose) at comparable concentrations do not reproduce these pathologic changes. Glucose reacts directly with singlet oxygen to form glucose radicals and other oxygen radical species [141, 142]. Under these conditions, EPA inhibited glucose-induced changes, including cholesterol crystals, in a concentration-dependent manner (Fig. 2). [131] The atorvastatin active metabolite also blocks lipid oxidation and cholesterol crystal biogenesis under hyperglycemic conditions that complements these actions of EPA. [127]

**Fig. 3:**
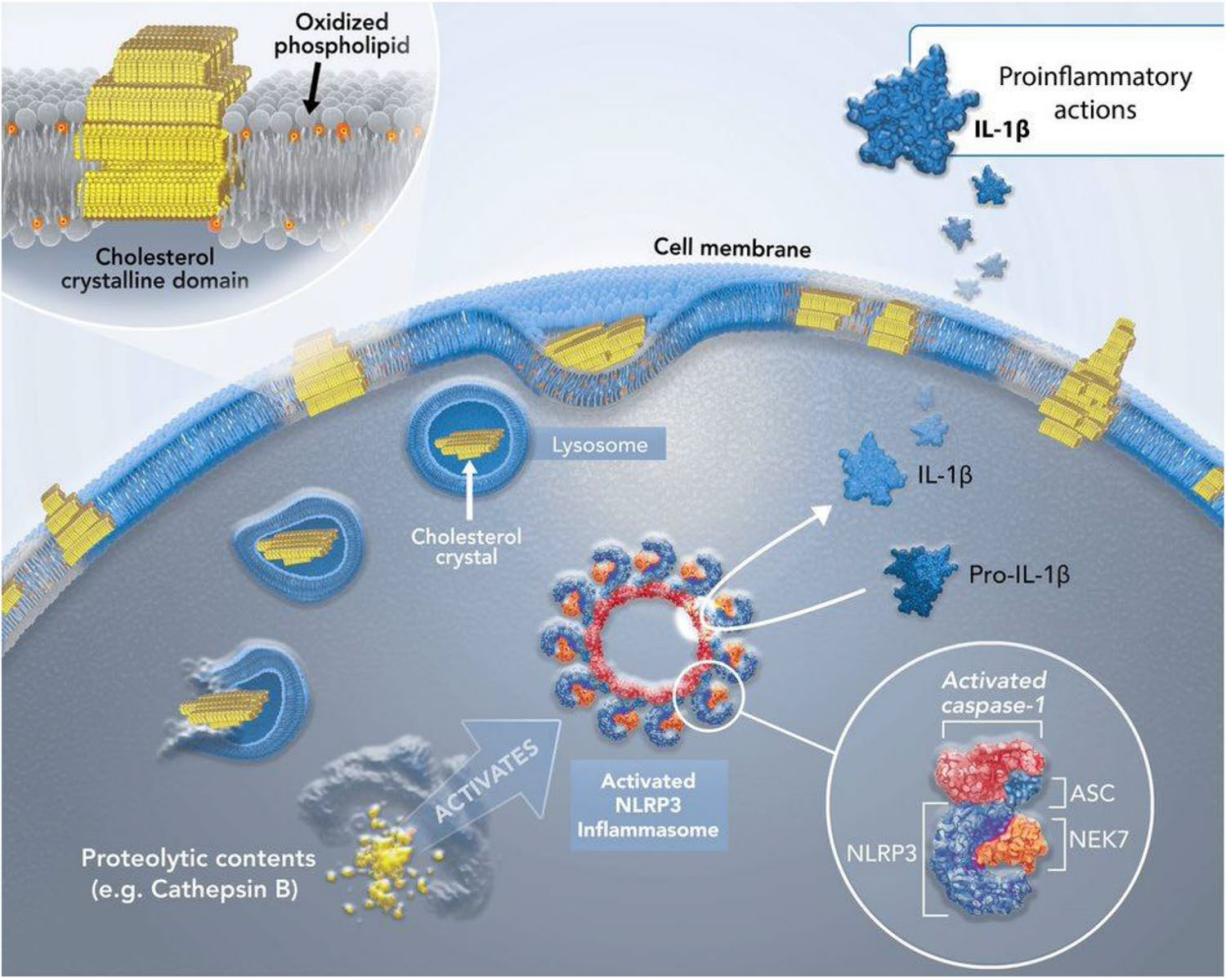
Cholesterol crystals can activate the NLRP3 inflammasome, leading to increased IL-1β release. Cholesterol crystals can enter cells via phagocytosis destined for lysosomal degradation. However, this process can cause lysosomal rupture and release of proteolytic contents into the cytosol (including cathepsin B and L). This leads to assembly of the (NOD-LLR and pyrin domain-containing protein 3 (NLRP3) inflammasome. NLRP3 recruits NIMA-related kinase 7 (NEK7), the adapted apoptosis-associated speck-like protein containing a caspase (ASC), and procaspase-1, which is then converted into activated caspase-1 which, in turn, converts the inactive pro- forms of interleukins (IL)-1β and IL-18 to their mature biologically functional forms for release

Cholesterol crystals also induce formation of neutrophil extracellular traps (NETs) in a manner dependent on oxygen radicals. NETs amplify and propagate thrombi in the arterial wall [143]. NETs also release mature IL-1β through NLRP3 inflammasome stimulation [144]. IL-1α associated with NETs also activates vascular endothelium [145]. NETs localize in lesions and genetic deletion of key NET production enzymes in ApoE-deficient mice exposed to a high-fat diet can reduce atherosclerosis to an extent similar to that observed in NLRP3-specific knockout animals [144]. This evidence strengthens the relationship or connection between cholesterol crystals and NLRP3-dependent inflammatory signaling within the advanced atheroma (Fig. 3).

## Omega-3 Fatty Acids and Statins Influence Endothelial Function

Impaired endothelium can promote the entry and retention of ApoB-containing lipoproteins in the arterial intima, triggering macrophage- and T cell-mediated inflammatory changes [146]. LDL-C modification or oxidation results in deamination of lysine residues on ApoB and formation of reactive aldehydes that are pro-inflammatory. Scavenger receptors on macrophages bind modified LDL, mediating its uptake and foam cell formation in the atheroma [147]. Activated macrophages sustain a local inflammatory reaction through release of cytokines and other signaling molecules [148, 149]. Increased risk for acute coronary and ischemic events as well as metabolic diseases are positively associated with levels of oxidized forms of circulating LDL [150–153]. Oxidized LDL-C also shifts endothelial conditions away from an anti-inflammatory and anti-thrombotic state as evidenced by increased production of pro-thrombotic mediators and vasoconstrictors, including endothelin-1, thromboxane A2 and prostaglandin H2.

Loss of endothelial function is a hallmark feature of atherothrombotic disease, leading to increased CV risk [154–156]. EC dysfunction begins with the fatty streak and incorporation of Apo B-containing particles such as LDL-C and, to a lesser extent, TGRLs, in the intima. Although they enter the intimal space at a lower rate, TGRLs efflux from the arterial wall at approximately 20-fold less than the rate of LDL-C, greatly increasing the exposure to oxidative damage and glycation to aggravate EC dysfunction [157]. Activated ECs express adhesion molecules promoting leukocyte (e.g, monocyte) attachment and transendothelial migration. These ECs also recruit immune cells including T cells and dendritic cells. The monocytes in the intima scavenge modified LDL-C and Apo E remnants. The activated macrophages not only promote inflammation, but also contribute to the plaque volume composed of cholesterol crystals, cholesteryl esters and cellular debris resulting from abnormal efferocytosis (clearance of dead cells.)

Additionally, ECs challenged with TNF-α and incubated with serum from subjects administered 4 g/d EPA had reduced expression of inflammatory biomarkers. The reductions in inflammatory signaling molecules were not reproduced in these human cells with serum from DHA-treated subjects [158]. In isolated arterial segments, EPA also reversed EC dysfunction under conditions of atherosclerosis with a statin [159]. The combination of EPA with a statin enhanced bioavailability of NO concomitant with reductions in nitroxidative stress, including peroxynitrite (ONOO^−^) generation. The comparative effects of n3-FAs were also evaluated in human ECs without exogenous challenges, in which EPA treatment improved the NO/ONOO^−^ release ratio and eNOS coupling efficiency. By contrast, neither DHA nor AA improved eNOS function under similar conditions [123]. These effects of EPA could be related to changes in total cellular fatty acid composition following treatment. Specifically, EPA treatment markedly increased levels of EPA and its immediate metabolic product, DPA, with no such changes in levels of DHA or AA in vitro. This resulted in a tenfold increase in the EPA/AA ratio, a predictor of CV risk and outcomes. Indeed, circulating EPA levels independently predicted event reduction in REDUCE-IT compared to other traditional risk factors like LDL-C and non-HDL. [55]

## Conclusion

**Fig. 4:**
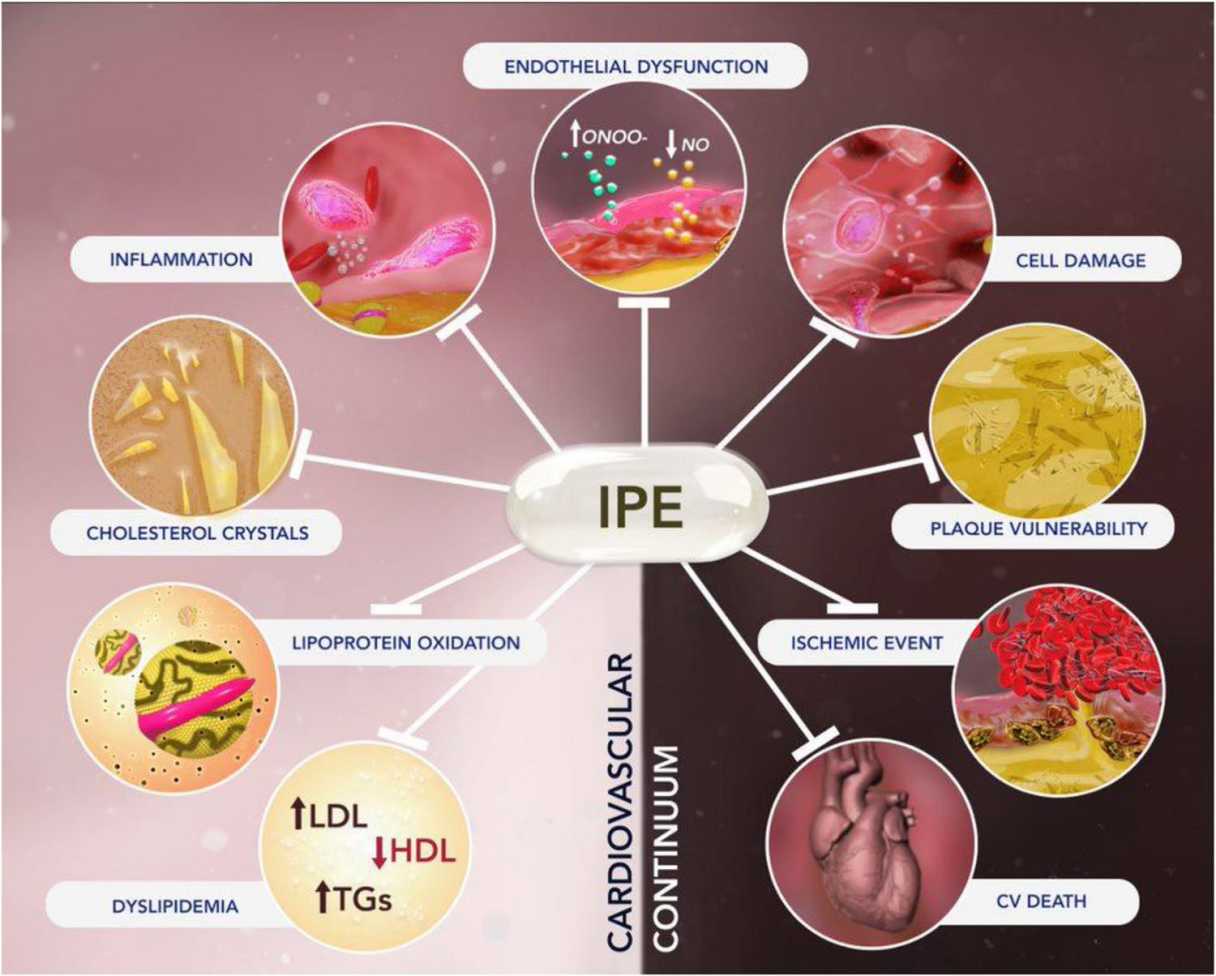
IPE interrupts the cardiovascular disease continuum at multiple points through broad pleiotropic mechanisms. The cardiovascular disease continuum progresses from early endothelial dysfunction and dyslipidemia to vascular inflammation that culminates in atherothromotic events, target organ damage, and death. The REDUCE-IT trial indicated that icosapent ethyl (IPE) treatment reduced a broad range of cardiovascular events by 25%. The active ingredient of IPE, EPA, may interrupt the cardiovascular continuum at several points, to limit overall risk

Among patients with metabolic disorders and CV disease, there remains residual risk despite effective oral and non-oral treatments that effectively lower LDL-C. Considerable evidence points to causality of TGRL in atherothrombosis, but risk reduction with strategies that reduce these lipids remain elusive. Despite pronounced reductions in TG levels, fibrates and the selective PPAR alpha modulator pemafibrate, along with various n3-FA formulations, did not reduce events in high-risk patients. However, two trials with EPA only formulations (i.e., IPE) showed reductions in CV events beyond TG lowering. These findings support a broad panel of pleiotropic actions as discussed above that associate with on-treatment EPA levels in REDUCE-IT that interfere with the CV disease continuum (Fig. 4). While not fully elucidated, these mechanisms of action for EPA include inhibition of thrombosis and platelet activation as well as preserved membrane structure, EC function, cholesterol distribution in the cell membrane and enhanced cholesterol efflux compared to DHA. In patients with atherosclerotic disease, EPA reduced plaque volume while increasing fibrous cap thickness when compared with statin treatment alone. As indicated by its abundance, DHA has critical roles in the functions of the central nervous system and retina, yet it may have limited effects on human atherothrombosis under the conditions studied. DHA containing formulations did not reproduce many of the benefits of IPE administration, likely related to differences in physicochemical properties, tissue distribution, and metabolism, or even by opposing certain mechanisms of EPA. Additional investigations into the molecular mechanism of action for EPA may yield new insights into the etiology and treatment of atherosclerosis.

## Data Availability

Data sharing not applicable to this article as no datasets were generated or analyzed in the current manuscript.
